# The Triple Win: The Influence of Institution-Sponsored Volunteering on Faculty and Staff Wellbeing and Satisfaction

**DOI:** 10.1007/s40670-026-02843-z

**Published:** 2026-08-07

**Authors:** Sarah-Ann Keyes, Alana D. Newell, Aimee K. Gardner

**Affiliations:** 1 School of Health Professions, Baylor College of Medicine, Houston, TX USA; 2 Baylor College of Medicine, Houston, TX USA; 3 University of Colorado Anschutz, Aurora, CO USA

**Keywords:** Volunteering, Self-determination, Employee well-being, Community, Institutional support

## Abstract

Employer-supported volunteering, including volunteer time-off (VTO), has been associated with positive work-related outcomes and may mitigate burnout. This report describes a five-year VTO initiative for faculty and staff at Baylor College of Medicine’s School of Health Professions. Leadership implemented targeted strategies to increase VTO participation and evaluated outcomes using time-off records and a post-program survey. VTO participation increased from 4% to 95%. Respondents reported high levels of community building, service learning, satisfaction, and organizational pride, with greater perceived benefits among faculty and individuals without prior volunteering experience. Self-determination theory was used as an analytic framework to interpret these findings.

## Background

Although volunteering is often viewed as a primarily altruistic activity, substantial evidence demonstrates physical and psychological benefits of volunteer engagement [1–3]. Even modest participation has been associated with improved mental health and psychological well-being [2, 3]. Employer-supported volunteering—through mechanisms such as adjusted work hours, recognition, use of institutional resources, and volunteer time-off (VTO)—aims to reduce barriers to participation while recognizing the reciprocal benefits of volunteering for both employees and organizations [4, 5].

Studies in corporate settings suggest that employees whose workplaces support volunteering report higher engagement, greater autonomy, and increased support from supervisors and colleagues [4]. Although VTO programs have been described in corporate settings and some universities have adopted similar policies, published evaluations of their implementation and impact in academic health professions institutions remain scarce [6]. Self-determination theory (SDT), a meta-theory of motivation and well-being, posits that optimal functioning occurs when individuals’ needs for autonomy, competence, and relatedness are met [7]. SDT has been used to explain how volunteering may enhance employee morale and satisfaction, particularly when individuals experience choice, skill development, and social connection through service [8–10].

The present study examines the impact of an institution-sponsored VTO program for health professions faculty and staff. SDT was used as an analytic framework to interpret observed outcomes of the program over time rather than as an a priori framework guiding program design.

## Activity

In 2015, a large health sciences university implemented an institution-wide VTO policy allowing eligible faculty and staff to engage in community service as paid time off during the work week. All benefit-eligible employees with at least one year of service were allotted one weekday per year for volunteering. While a list of preferred community organizations was provided, participants were free to select service opportunities aligned with their interests, values, and availability, including a team walk to raise money for Alzheimer’s research and an organized team to provide meals to underserved children in the greater Houston Area.VTO utilization was tracked through the institution’s centralized human resources system.

Beginning in 2018, the School of Health Professions (SHP) incorporated VTO utilization into a five-year continuous quality improvement initiative with an aspirational goal of achieving 100% participation among faculty and staff after it was noted that there was almost no participation in the institution-wide policy from 2015 to 2018. Participation data were reviewed annually, and leadership adjusted communication and implementation strategies in response to observed trends (Table 1). Interventions included general and targeted email outreach, encouragement during mentoring discussions, and the introduction of institutionally organized group volunteer activities.

**Table 1:** Interventions and participation rates

Year	Intervention	Eligible Faculty/Staff (*N*)	Participants (*n*)	Participation Rate
1	Program was conceptualized	30	1	4%
2	Generic email distributions & encouraged participation during mentoring discussions	32	0	0%
3	Generic email distributions; encouraged participation during mentoring discussions; organized two group activities	32	24	76%
4	Generic email distributions; encouraged participation during mentoring discussions; organized three group activities	33	30	90%
5	Generic email distributions; focused email distributions; encouraged participation during mentoring discussions; organized three group activities	38	36	95%

At the conclusion of the five-year initiative, and following Institutional Review Board approval (H-42599), SHP faculty and staff were invited to complete an anonymous online survey. The survey assessed attitudes toward VTO participation, perceived benefits, prior volunteering experience, frequency of VTO use, time since most recent volunteering, and barriers to participation. Twenty-two items measured reactions across four domains—Community Building, Service Learning, Satisfaction, and Organizational Pride—using a five-point Likert scale (1 = strongly disagree; 5 = strongly agree) (Table 2). Participants also reported role (faculty or staff), preferences for group versus individual volunteering, and perceptions of the ideal number of VTO days.

**Table 2:** Means and percentage strongly agreed for survey items by category based on 1–5 likert scale

Category	Item	Mean (SD)	% Agreed or Strongly Agreed
Learning	Participating in the VTO program allowed me to learn more about non-profits	4.34 (0.90)	85%
	Participating in the VTO program allowed me to learn more about community needs	4.48 (0.74)	93%
	Participating in the VTO program allowed me to expand my awareness of services available in the community	4.62 (0.62)	93%
	Participating in the VTO program allowed me to broaden my awareness of underserved populations in the community	4.28 (0.75)	83%
	I learned new knowledge or skills through my volunteering experience	4.10 (0.90)	72%
Community Building	Participating in the VTO program allowed me to spend time with faculty/staff in the School with whom I don’t frequently interact	4.54 (0.71)	88%
	Participating in the VTO program allowed me to learn more about faculty/staff in the School with whom I don’t frequently interact	4.50 (0.76)	85%
	Participating in the VTO program helped create a sense of social connectedness with faculty/staff who also participated in that event.	4.54 (0.71)	89%
	Participating in the VTO program helped me expand my personal and professional network	4.03 (0.94)	66%
	Participating in the VTO program helped me learn from and develop relationships with those in the community	4.17 (1.00)	72%
Satisfaction	Participating in the VTO program has helped me feel good about myself	4.72 (0.53)	97%
	Participating in the VTO program has helped increase feelings of self-worth	4.62 (0.62)	93%
	Participating in the VTO program has helped me feel like I am contributing to the greater good	4.76 (0.44)	100%
	Participating in the VTO program helps me feel like I am being true to myself	4.45 (0.69)	90%
	Participating in the VTO program helps me show more of my whole self at work	4.55 (0.83)	85%
Organizational Pride	Being able to see how my volunteering benefitted the community has made me proud of being an employee at this organization	4.66 (0.61)	93%
	Participating in the VTO program helped me feel pride for working at this organization	4.66 (0.61)	93%
	I enjoyed representing this organization outside of the workplace	4.68 (0.61)	93%
	I enjoyed being able to promote this organization’s values outside of work	4.66 (0.61)	93%
	Being able to volunteer through the VTO program has helped align my values and those of this organization	4.34 (0.81)	79%
	I was proud to tell my family and/or friends about my volunteering experience	4.66 (0.67)	90%
	Opportunities like VTO are one of the reasons why I believe this organization is a great place to work	4.52 (0.69)	90%

Descriptive statistics, independent samples t-tests, and analyses of variance were conducted using SPSS version 28.0.

## Results

As shown in Table 1, participation in the VTO program was minimal during the first two years of the initiative, with only one faculty member participating in year one (4%) and no participation in year two. Following the introduction of organized group volunteer activities in year three, participation increased substantially to 76%, continuing to rise to 90% in year four and 95% in year five.

87% (33/38) of faculty and staff completed the year five survey. Most respondents (70%) reported no volunteering prior to adoption of the VTO program. Among those with prior experience, reported prior volunteering ranged from one to two days annually to more than ten days. Over half of respondents (64%) reported no barriers to VTO use. The most commonly cited barrier was finding time (24%), followed by difficulty identifying a volunteer site (6%), finding others to volunteer with (3%), and logging events in the human resources system (3%).

Most respondents (78%) reported using VTO within the previous nine months. A majority preferred group volunteering (61%), while 26% were neutral and 13% preferred individual volunteering. Respondents generally favored additional VTO, with 45% identifying four VTO days per year as the ideal allotment; nearly all indicated they would volunteer more if additional VTO were available.

Across domains, respondents reported high perceived benefits, with 85% agreeing or strongly agreeing with items related to Community Building and Service Learning, 90% for Satisfaction, and 85% for Organizational Pride (Table 2). Faculty reported greater personal satisfaction from volunteering than staff (4.81 vs. 4.30, *p* = 0.040), whereas perceptions of Community Building (4.70 vs. 4.33, *p* = 0.245), Service Learning (4.48 vs. 4.31, *p* = 0.556), and Organizational Pride (4.77 vs. 4.50, *p* = 0.274) did not differ significantly between groups.

**Fig. 1:**
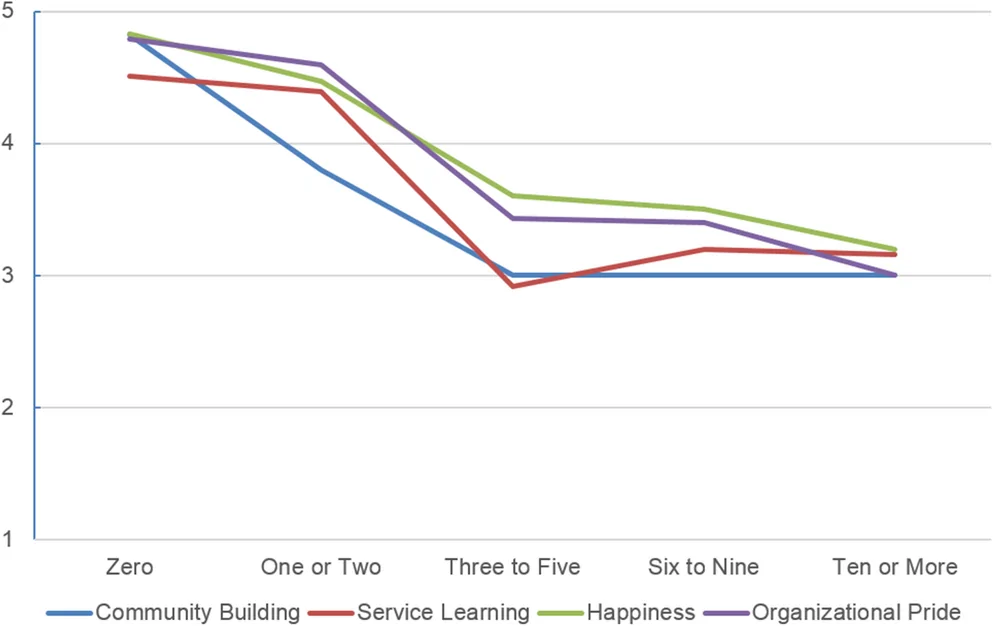
Participant ratings of benefits and years of experience

No differences emerged based on volunteer frequency during the initiative or time since the most recent volunteering episode. However, individuals without prior volunteering experience reported significantly higher benefits across all four domains compared with those who had volunteered previously (all *p* ≤ 0.005). Benefits decreased as participants’ self-reported volunteering frequency prior to the VTO program increased, plateauing among those who had volunteered six or more times annually before participating in the program (Fig. 1).

## Discussion

Understanding effective approaches to employer-supported volunteering may provide academic health professions institutions with additional opportunities to support employee well-being. The positive outcomes observed in this study suggest that SDT offers a useful lens for interpreting how VTO programs may function in this context.

The program remained voluntary throughout the five-year period and continues in the present, preserving employee choice and supporting autonomy. Although leadership actively promoted participation, faculty and staff retained discretion over whether and how to engage. Respondents also reported learning new skills and developing social connections, aligning with the SDT constructs of competence and relatedness. Relatedness appeared particularly salient, as participation increased markedly following the introduction of organized group events and most respondents expressed a preference for group volunteering.

Individuals without prior volunteering experience reported greater perceived benefits than those who had volunteered previously, suggesting potential diminishing returns for highly experienced volunteers. These findings indicate that employer-supported volunteering may be especially impactful for engaging individuals who are not already involved in service activities.

Although participation increased following the introduction of organized group volunteer activities, motivations for engagement are likely multifactorial and warrant further study. Future research should examine how specific program features interact with autonomy, competence, and relatedness to influence participation. Additional studies could also evaluate whether increasing institutionally provided VTO days further enhances volunteer participation and faculty satisfaction while accounting for the influence of organized group volunteer activities. Finally, the relationships among institutional volunteering, organizational pride, and faculty satisfaction warrant further exploration. Finally, we cannot determine why satisfaction plateaued for faculty with former volunteering experienced. This may reflect diminished novelty, differences in motivation, or other factors and warrants further investigation.

In summary, this study provides initial support for institution-sponsored volunteering as a strategy to enhance faculty and staff well-being in health professions education. Programs that preserve choice while fostering learning and connection may be particularly beneficial, especially for those new to volunteering.

## Declarations

### Disclaimers

No disclaimers.

### Previous Presentations

September 15, 2023; Houston, Texas.
